# RPS: a comprehensive database of RNAs involved in liquid–liquid phase separation

**DOI:** 10.1093/nar/gkab986

**Published:** 2021-10-28

**Authors:** Mengni Liu, Huiqin Li, Xiaotong Luo, Jieyi Cai, Tianjian Chen, Yubin Xie, Jian Ren, Zhixiang Zuo

**Affiliations:** School of Life Sciences, State Key Laboratory of Oncology in South China, Cancer Center, Collaborative Innovation Center for Cancer Medicine, Sun Yat-sen University, Guangzhou 510060, China; School of Life Sciences, State Key Laboratory of Oncology in South China, Cancer Center, Collaborative Innovation Center for Cancer Medicine, Sun Yat-sen University, Guangzhou 510060, China; School of Life Sciences, State Key Laboratory of Oncology in South China, Cancer Center, Collaborative Innovation Center for Cancer Medicine, Sun Yat-sen University, Guangzhou 510060, China; School of Life Sciences, State Key Laboratory of Oncology in South China, Cancer Center, Collaborative Innovation Center for Cancer Medicine, Sun Yat-sen University, Guangzhou 510060, China; School of Life Sciences, State Key Laboratory of Oncology in South China, Cancer Center, Collaborative Innovation Center for Cancer Medicine, Sun Yat-sen University, Guangzhou 510060, China; Precision Medicine Institute, The First Affiliated Hospital, Sun Yat-sen University, Guangzhou 510060, China; School of Life Sciences, State Key Laboratory of Oncology in South China, Cancer Center, Collaborative Innovation Center for Cancer Medicine, Sun Yat-sen University, Guangzhou 510060, China; School of Life Sciences, State Key Laboratory of Oncology in South China, Cancer Center, Collaborative Innovation Center for Cancer Medicine, Sun Yat-sen University, Guangzhou 510060, China

## Abstract

Liquid–liquid phase separation (LLPS) is critical for assembling membraneless organelles (MLOs) such as nucleoli, P-bodies, and stress granules, which are involved in various physiological processes and pathological conditions. While the critical role of RNA in the formation and the maintenance of MLOs is increasingly appreciated, there is still a lack of specific resources for LLPS-related RNAs. Here, we presented RPS (http://rps.renlab.org), a comprehensive database of LLPS-related RNAs in 20 distinct biomolecular condensates from eukaryotes and viruses. Currently, RPS contains 21,613 LLPS-related RNAs with three different evidence types, including ‘Reviewed’, ‘High-throughput’ and ‘Predicted’. RPS provides extensive annotations of LLPS-associated RNA properties, including sequence features, RNA structures, RNA–protein/RNA–RNA interactions, and RNA modifications. Moreover, RPS also provides comprehensive disease annotations to help users to explore the relationship between LLPS and disease. The user-friendly web interface of RPS allows users to access the data efficiently. In summary, we believe that RPS will serve as a valuable platform to study the role of RNA in LLPS and further improve our understanding of the biological functions of LLPS.

## INTRODUCTION

Liquid-liquid phase separation (LLPS) is a reversible process driving the formation of membraneless organelles (MLOs) such as nucleoli, P-bodies and stress granules (SGs). During the LLPS process, many biomolecules including protein and RNA aggregate together into biomolecular condensates, which play critical roles in the regulation of many biological processes such as cellular stress responses (1), homeostasis maintenance (2) and development (3). Meanwhile, increasing evidence has shown that the dysregulation of LLPS is closely associated with a variety of diseases such as amyotrophic lateral sclerosis (ALS) (4–6), frontotemporal dementia (FTD) (4–6), Alzheimer's disease (AD) (7), cancer (8), as well as infectious diseases (9).

Deciphering the components of LLPS is vital to understand the roles of LLPS in physiological and pathological processes. Previous studies primarily focused on the protein components of LLPS. It has been demonstrated that G3BP protein was required for SG formation in mammalian cells during oxidative stress (10), and the MEG1 and MEG3 proteins are required for P-granule formation in C. elegans (11). These LLPS proteins generally contain intrinsically disordered regions (IDRs) or low-complexity domains (LCDs), which contribute to phase separation via multivalent weak interactions (12). To facilitate the study of proteins involved in LLPS, several databases have been developed to record proteins associated with LLPS, such as LLPSDB (13), PhaSePro (14), PhaSepDB (15), DrLLPS (16), RNAgranuleDB (17) and HUMAN CELL MAP (18).

In recent years, emerging evidences have proved that RNA also has fundamental roles in the regulation of LLPS. It has been recognized that RNAs act as a buffer in the nucleus where high RNA concentrations keep RBPs soluble (19). Besides, RNA can phase separate without protein and promote or inhibit phase separation (5). Similar to proteins, RNA can also serve as a seed for biomolecular assemblies, such as lncRNA NEAT1, which functions as a scaffold by interacting with other RBPs for paraspeckles construction (20). In addition, numerous studies have suggested that RNA-dependent condensates are tightly regulated by various RNA properties, such as RNA sequence, structure, RNA modifications, RNA–RNA interactions and RNA–protein interactions. For example, Khong *et al.* (1) suggested that relatively long transcripts are preferentially involved in SGs formation, which harbors more sites for possible interactions with RNA-binding proteins (RBPs) and/or RNAs. Moreover, compared with less structured RNAs, highly structured RNAs can rearrange the composition of protein aggregates for having more interactions with proteins (21). G-quadruplex (GQ), a specific RNA tertiary structure motif, can trigger RNA phase separation under physiological conditions in vitro (22). Other features like RNA modifications are also critical in phase separation. For instance, N6-methyladenosine (m^6^A), as the most prevalent mRNA modification, was considered as a multivalent scaffold for binding YTHDF proteins in mammalian cells (23). Furthermore, RNA expression levels are essential for the formation and maintenance of condensates. A recent study of condensate transcriptome has revealed that SGs assembly relied on the condensation of poorly translated mRNAs in mammalian and yeast cells (1). Of note, it is reported that mutations can impact RNA-dependent LLPS. For example, the disease-causing G4C2 repeat expansion in the C90orf72 gene has been shown to mediate LLPS both in vitro and in vivo (5,24). Collectively, these features encoded in RNA confer specific condensate biophysical properties, which are essential for condensate functions in homeostasis.

**Figure 1.**
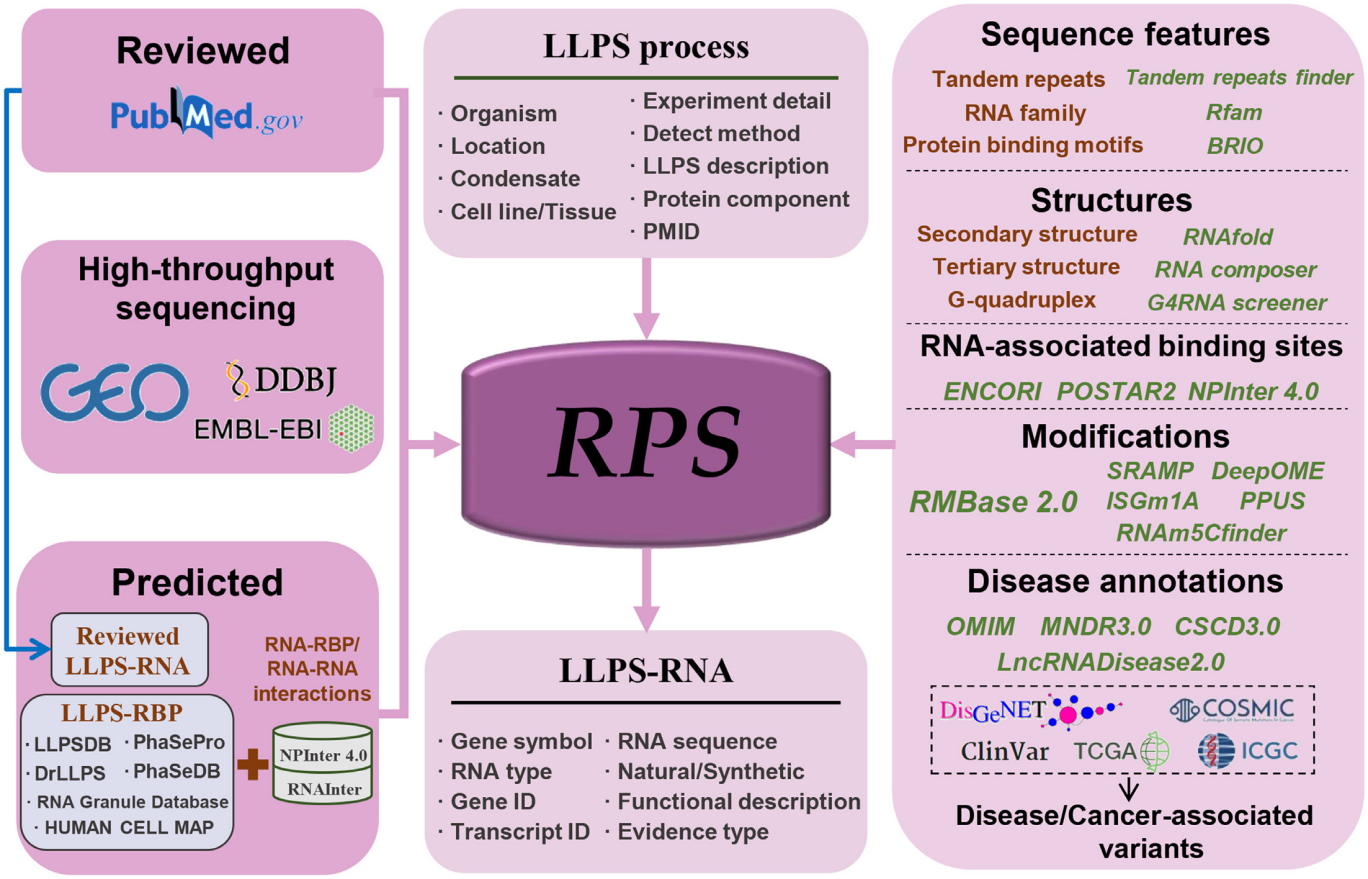
Overall design and construction of RPS. LLPS-related RNAs deposited in RPS were derived from literature mining, high-throughput sequencing experiments and predictions based on interaction networks. RPS provides detailed information of LLPS-related RNAs and LLPS processes. In addition, RPS contains extensive annotations of RNA properties for each LLPS-related RNA, such as RNA sequence, RNA structure, RNA-RBP interactions, RNA–RNA interactions, RNA modifications, as well as disease annotations.

Although numerous studies have paid attention to the role of RNAs in LLPS, an integrative data resource of LLPS-related RNAs is still not available. To fill this gap, we present RPS (http://rps.renlab.org), a comprehensive database of LLPS-related RNAs in 20 distinct biomolecular condensates across eukaryotes and viruses. RPS contains 21 613 LLPS-related RNAs derived from literature mining, high-throughput analysis and predictions based on interaction network (Figure 1). RPS provides basic information of LLPS-related RNAs and LLPS processes, as well as plentiful annotations of RNAs, including sequences, RNA secondary structures, RNA–RNA/RBP binding sites and modifications. Additionally, disease annotations of LLPS-related RNA are also available. With these features, we anticipate that RPS will be helpful to investigate the role of RNA in LLPS and provide new insights into human disease.

## MATERIALS AND METHODS

### Data collection

RPS collected RNA-associated LLPS data with three evidence types. (i) Reviewed: The literature mining was performed by searching NCBI PubMed using keywords combinations ‘((phase separation[Title/Abstract]) OR (phase transition[Title/Abstract]) OR (biomolecular condensates[Title/Abstract]) OR (MLOs[Title/Abstract]) OR (membraneless organelle[Title/Abstract])) AND (RNA)’. We retrieved a total of 1507 papers published up to 30 June 2021, then screened out 131 publications that describe biomolecular condensates and related RNAs by rechecking abstracts manually. Both natural and synthetic RNAs with this evidence type were curated. (ii) High-throughput: RNA composition of MLOs under different conditions can be identified by RNA sequencing methods. By retrieving related keywords (same with ‘Reviewed’ type) from the GEO (25), EMBL-EBI (26) and DDBJ (27) databases, we collected seven MLO-associated datasets (including 82 samples) from humans, mice and yeast (Table S1). Further analyses were performed on these datasets to identify RNAs that participate in the formation of MLOs. (iii) Predicted: RNA–protein and RNA–RNA interactions underlie the ability of particular RNAs to undergo LLPS (28). Therefore, we anticipated that RNA interactors with known-related RNAs/RBPs are likely to undergo LLPS. Briefly, we obtained LLPS-related proteins from six resources, including LLPSDB (13), PhaSePro (14), PhaSepDB (15), DrLLPS (16), RNAgranuleDB (17) and Human cell Map (18). Then, we filtered proteins that were only relevant with RNA-free condensates (such as receptor cluster and Z granule) and retained 1766 LLPS-related proteins for prediction. After that, based on experimentally validated RNA-associated interactions obtained from NPInter 4.0 (29) and RNAInter (30), we identified 1358 RNA interactors with these retained LLPS-related proteins and ‘Reviewed’ LLPS-related RNAs.

### Analysis of RNA-Seq datasets

All collected RNA-seq datasets were processed with a uniform pipeline described below. Raw reads were first trimmed by TrimGalore (v2.10, https://github.com/FelixKrueger/TrimGalore) to remove low-quality bases (Phred score < 25) and adapters contamination. The trimmed reads were aligned to the reference genome (human: GRCh38; mouse: GRCm38; yeast: R64-1-1) using STAR (v.2.7.6) with default parameters (31) and further counted by featureCounts (v.2.0.1) (32). After that, we performed pairwise comparisons between the condensate group versus the control group (e.g. RNA granule vs cytoplasm) or condensate groups under distinct conditions (e.g. heat shock-induced RNA granule vs unstressed RNA granule). Normalization and differential expression analysis were then performed using DESeq2 (v1.28.1) (33). To determine phase-separated condensates enriched transcripts, we calculated the fold enrichment over the control group and applied a threshold of two-fold enrichment with a *P*-value <0.05. RNA transcripts that meet this criterion were considered to be enriched in this condensate.

### Curation of LLPS processes

We extracted detailed information of the LLPS system, including detect methods, such as fluorescence recovery after photobleaching (FRAP), electron microscopy (EM), etc., experiment conditions such as RNA/salt concentrations, salt, pH, pressure, and temperature, protein components. It should be noted that ‘Predicted’ LLPS-related RNAs are not assigned to a particular LLPS process, therefore having no corresponding LLPS information.

### Annotation of LLPS-related RNAs

RPS provides various basic information of LLPS-related RNAs, such as gene symbol, gene ID, transcript ID, sequence, RNA type, functional description. These data were preferentially extracted from the original publications, otherwise, they were acquired from Ensembl (34) or UCSC (35) databases. For part ‘reviewed’ LLPS-related RNAs, sequences were curated from the original publications. Other RNA sequences were either represented by corresponding canonical transcripts obtained via UCSC table browser or obtained from existed databases, including LNCipedia (36), miRbase (37) and NONCODE (38).

To improve our understanding of how RNA contributes to phase separation, RPS integrated multiple resources and tools to annotate distinct LLPS-associated properties of RNAs. Sequence-specific features, including tandem repeats and RNA sequence families, were detected separately using tandem repeat finder (39) and Rfam web server (40). For structure annotation, we first extracted information of LLPS-associated structures from the original publications. Then, we predicted RNA secondary structures based on a minimum free energy (MFE) algorithm using RNAfold (v2.4.1) from ViennaRNA package 2.0 (41). For RNAs within 500 nt, we further used RNAComposer (42) to assemble the 3D modeling according to their predicted secondary structures. GQ structures were predicted using the G4RNA screener web server (43) with the default settings. Moreover, the experimentally verified and predicted RNA–protein and RNA–RNA binding sites were acquired from ENCORI (44), POSTAR2 (45) and NPinter 4.0 (29). Besides, we identified known sequence and secondary structure protein binding motifs in RNAs from humans and mice via BRIO web server (46). As another LLPS-associated property, RNA modification can also potentially contribute to the features of native condensate. In addition to integrating the RNA modifications sites with experimental validation from RMBase 2.0 (47), we employed SRAMP (48) for m^6^A sites prediction, DeepOME (49) for 2′-O-methylation (Nm) sites prediction, RF-PseU (50) and PPUS (51) for pseudouridine (Ψ) sites prediction, RNAm5Cfinder (52) for 5-methylcytidine (m^5^C) sites prediction and ISGm1A for N^1^-methyladenosine (m^1^A) sites prediction.

To discover potential relations between LLPS-related RNAs and diseases, we collected RNA-disease associations with experimental evidence from OMIM (53), DisGeNET (54), MNDR 3.0 (55), LncRNADisease 2.0 (56) and CSCD 2.0 databases (57). Furthermore, we also integrated disease-associated variants from DisGeNET (54) and ClinVar (58), as well as cancer-associated variants taken from COSMIC (59), ICGC (60) and TCGA (61). In RPS, we considered RNAs having RNA-disease associations or disease/cancer-associated variants as disease-associated RNAs. The genomic coordinates of all data resources were further converted to GRCh38 or GRCm38 using the LiftOver program (35).

### Classification of phase-separated condensates

To better curate the deposited data, we categorized all biomolecular condensates into three classes: (i) nucleus, including paraspeckle, Cajal body, DNA damage foci, histone locus body, nuclear body, nuclear speckle, nucleolus, PML nuclear body, PcG body; (ii) cytoplasm: centrosome, P-body, cytoplasmic granule, G body, neuronal granule, stress granule and TIS granule; (iii) others, including germ cell condensates (P granule, Nuage and Balbiani body) and RNP granules in unknown locations, as well as coacervate droplets. These phase-separated condensates were further annotated with Gene Ontology (GO) cellular component terms.

### Database and web interface implementation

All data in RPS were stored and managed by MySQL tables. The server-backend development was based on java and the web-frontend interfaces were implemented in Hyper Text Markup Language (HTML), Cascading Style Sheets (CSS) and JavaScript (JS). In order to present data more efficiently and intuitively, multiple statistical diagrams were embedded in the website. The interactive heat maps showing the expression abundance and differential expression were

constructed by Ant Design toolkit. The boxplots showing the differential expression and the charts presenting the interaction network of LLPS-related RNAs were drawn by Echarts. The RNA tertiary structures are displayed using 3Dmol.js (62). Furthermore, RPS implemented a genome browser to present genomic annotations using UCSC Genome Browser (http://genome.ucsc.edu/) (35).

## RESULTS

### Database content

**Figure 2.**
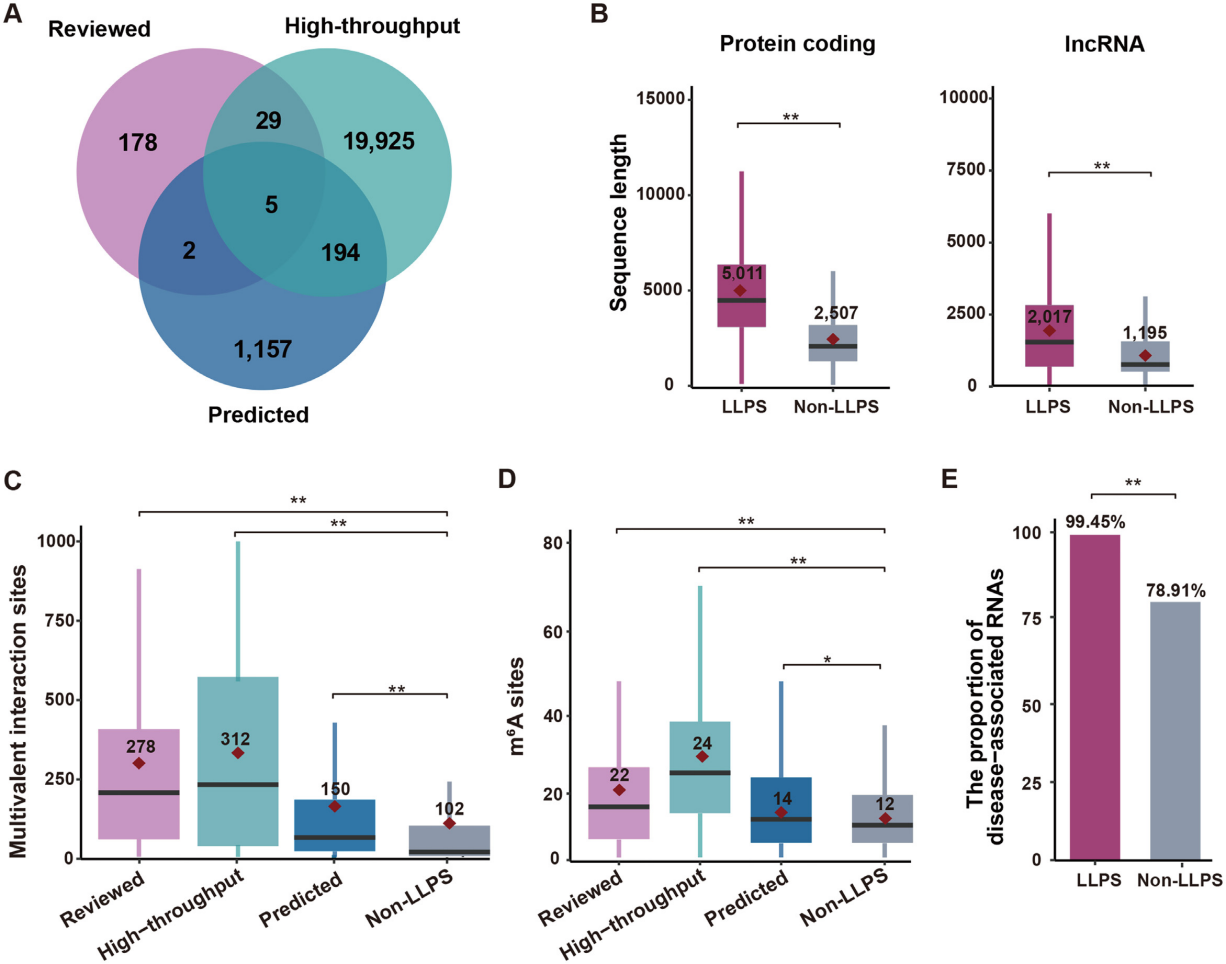
Statistical analysis of LLPS-RNAs in RPS. (**A**) The overlap between LLPS-related RNAs with different evidence types. (**B**) Comparison of sequence lengths of LLPS-related RNAs and non-LLPS-related RNAs for protein-coding RNA and lncRNA, separately. Wilcoxon signed-rank test, ** *P*-value < 0.01. (**C**) Comparison of the multivalent interaction sites (RNA–RNA interaction and RNA-RBP interaction) of LLPS-related RNAs and non-LLPS-related RNAs in humans. Wilcoxon signed-rank test, ** *P*-value < 0.01. (**D**) Comparison of experimentally verified m^6^A sites of LLPS-RNAs and non-LLPS-related RNAs in humans. (**E**) The proportion of disease-associated RNAs in LLPS-related RNAs and non-LLPS-related RNAs. Two-proportions *z*-test, ** *P*-value < 0.01.

Currently, RPS contains 42 417 entries for 21 613 unique LLPS-related RNAs from 13 organisms. There are three different evidence types of LLPS data deposited in RPS: (i) ‘*Reviewed’*: 523 entries for 337 RNAs were validated to participate in LLPS through *in vitro* or *in vivo* experiments. Among them, 328 entries are from 214 natural RNAs. The other 123 synthetic nucleotides with 195 entries were designed for condensate reconstruction experiments, such as homotypic RNA polymers. (ii) ‘*High-throughput*’: 20 153 RNAs involved in 18 different LLPS processes were identified by high-throughput analyses. These RNAs were considered as RNA components of MLOs such as SGs, paraspeckles, P-body, G-body and other RNA granules (Table S1). (iii) ‘*Predicted*’: 1358 RNAs were predicted as potential LLPS-related RNAs based on RNA-associated interactiozns, where the transcripts of NEAT1, BRCA1, NORAD, MALAT1 and ACTB were supported by three types of evidence. Besides, total 199 ‘Predicted’ LLPS-related RNAs were validated either by ‘Reviewed’ or ‘High-throughput’ evidence (Figure 2A), demonstrating the validity of the interaction-based prediction method. Regarding the subcellular localization, 20 598 RNAs are localized in the cytoplasm, 1943 RNAs participate in the formation of biomolecular condensates in nuclear, and 369 RNAs belong to other condensates in germ cells or unknown locations (Table 1). The majority of LLPS-related RNAs are derived from humans, mice and yeast. Besides, protein-coding RNA and lncRNA are the most abundant RNA types stored in RPS (Table 2). In addition, RPS provides a plentiful of annotations for the LLPS-related RNAs, such as RNA–RNA/RBP binding sites, RNA modification sites and disease-associations.

**Table 1.** LLPS-related RNAs in distinct condensates with different evidence types

Condensate	Location	Reviewed	High-throughput	Predicted	Total
Cytoplasmic granule	Cytoplasm	0	10 788	3	10 791
P-body	Cytoplasm	23	8431	747	9080
Stress granule	Cytoplasm	35	4736	954	5662
Paraspeckle	Nucleus	52	787	67	903
Nucleolus	Nucleus	5	0	791	796
Coacervate droplet	Others	174	0	163	336
G body	Cytoplasm	5	238	0	243
Nuclear speckle	Nucleus	1	0	242	242
TIS granule	Cytoplasm	6	0	106	112
Nuclear body	Nucleus	16	0	75	91
PML nuclear body	Nucleus	0	0	33	33
P granule	Others	28	0	3	31
Cajal body	Nucleus	1	0	2	3
DNA damage foci	Nucleus	1	0	2	3
Histone locus body	Nucleus	1	0	1	2
Neuronal granule	Cytoplasm	0	0	2	2
Nuage	Others	0	0	2	2
Balbiani body	Others	0	0	1	1
Centrosome	Cytoplasm	0	0	1	1
PcG body	Nucleus	0	0	1	1

**Table 2.** LLPS-related RNAs of different RNA types from distinct organisms

RNA type	*Homo sapiens*	*Mus musculus*	*Saccharomyces cerevisiae*	Other eukaryotes	Virus	Synthetic
protein_coding	7269	6250	1928	33	0	0
lncRNA	2116	1002	0	0	0	0
pseudogene	738	366	58	0	0	0
miRNA	422	47	0	2	8	0
snoRNA	20	50	55	0	0	0
snRNA	31	22	0	0	0	0
virus ssRNA	0	0	0	0	13	0
others	219	835	8	2	0	0
synthetic nucleotides	0	0	0	0	0	123

### Characteristics of LLPS-related RNAs

To characterize the LLPS-related RNAs, we systematically compared several RNA properties of LLPS-related and non-LLPS-related RNAs in humans. The non-LLPS-related RNAs were defined as human canonical transcripts that were not deposited in RPS. Consistent with previous studies (1,63), the sequences of LLPS-related RNAs are significantly longer than those of non-LLPS-related RNAs for both types (Figure 2B, *P*-value < 0.01, Wilcoxon signed-rank test), indicating the promoting effect of RNA length on LLPS. In addition, comparisons of multivalent interaction sites (RNA–RBP and RNA–RNA binding sites) showed that LLPS-related RNAs of all three evidence types have significantly more interactions sites than those in non-LLPS-related RNAs (Figure 2C, *P*-value < 0.01, Wilcoxon signed-rank test). This suggests that increased multivalent interactions are likely to promote LLPS, which is in line with previous findings (64). Moreover, LLPS-related RNAs have significantly more experimentally verified m^6^A sites than non-LLPS-related RNAs (Figure 2D), which is consistent with previous findings that m^6^A plays a critical role in driving LLPS in mammalian cells (23). In term of the relevance to disease, LLPS-related RNAs show a higher proportion of disease-associated RNAs than that in non-LLPS-related RNAs (Figure 2E, *P*-value < 0.01, two-proportions *z*-test), indicating that RNA-mediated phase separation is closely related to human diseases.

### Web interface and usage

**Figure 3.**
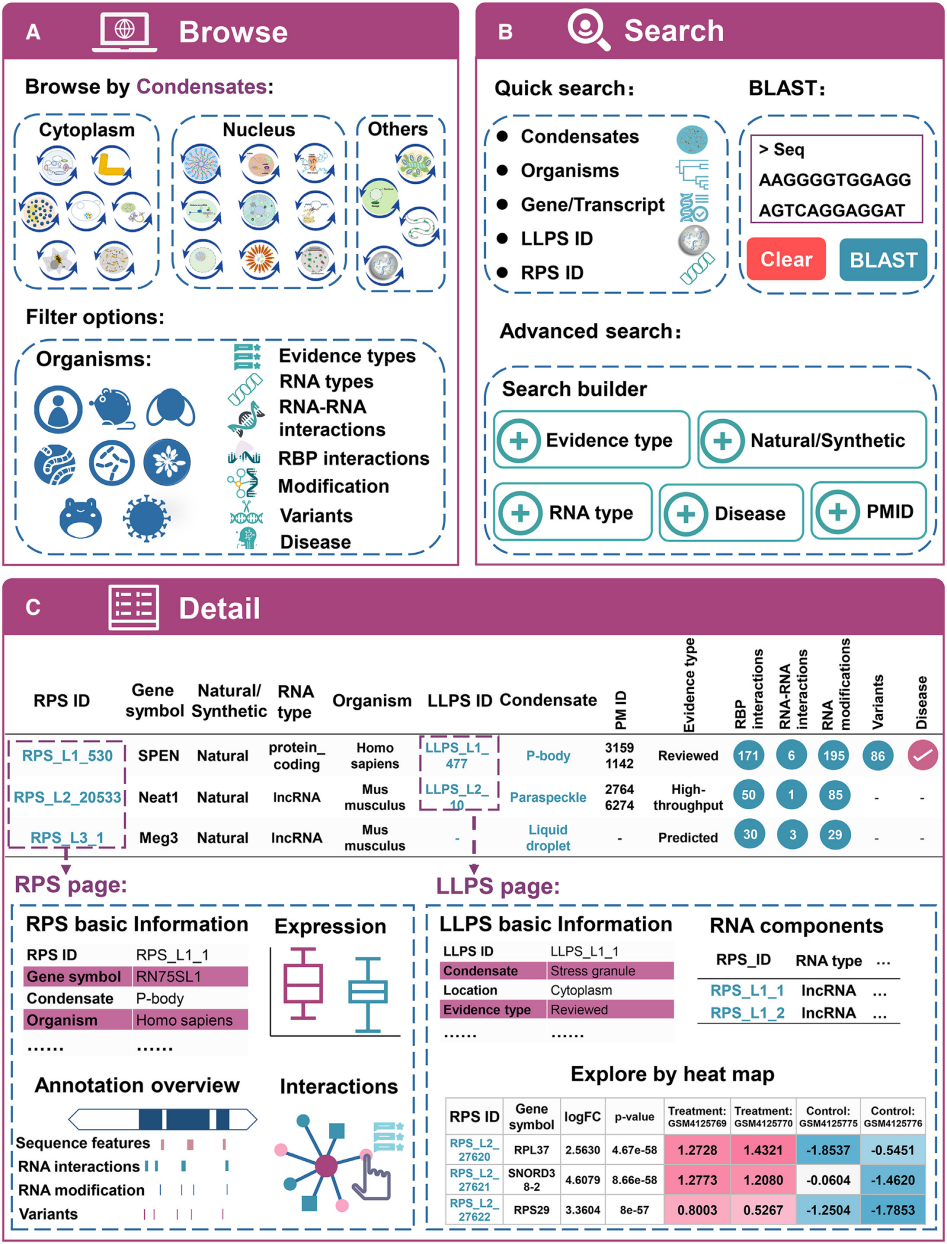
Basic functions of RPS web interface. (**A**) The browsing interface of RPS. (**B**) The main modules of search interface in RPS. (**C**) The detailed information for LLPS-related RNA and LLPS process can be linked to the RPS detail page and the LLPS detail page via clicking RPS ID and LLPS ID, separately.

RPS provides a user-friendly web interface, allowing users to explore LLPS-related RNAs or processes of interest easily and interactively (Figure 3).

*Browse*, users can browse the entries by condensates. For each condensate, the browse page presents a statistical chart, as well as diagrams for the LLPS-related RNAs distribution in distinct organisms and RNA types. The query results can be filtered by extra options, including Organism, Evidence type, RNA type and other RNA features (Figure 3A).

*Search*, RPS provides two ways to search the database (Figure 3B). First, an advanced search engine is developed to enable precise queries, providing various options including Gene symbol, Gene ID, Transcript ID, Organism, Condensate, Evidence type, RNA type, Natural/Synthetic, RPS ID, LLPS ID and PMID. Second, users can perform a sequence similarity search against RPS by an online BLAST server (v 2.7.1), which was implemented on the ‘BLAST’ page.

*Detail*, the details of each entry are displayed on the ‘Detail’ page by clicking any RPS ID or LLPS ID (Figure 3C). The detail for a LLPS-related RNA contains the evidence that it participates in LLPS, annotations of various RNA properties such as sequence features, structures, RNA–protein/RNA interactions, and RNA modifications, and annotations of disease associations. Furthermore, RPS allows users to browse genomic features of interested RPS entries by integrating all the RPS data into the UCSC genome browser. The detail page of the LLPS processes records experiment conditions, detecting methods, description of phase behavior, protein components and RNA components. Notably, an interactive heatmap was implemented explicitly for a high-throughput experiments-derived LLPS process on the LLPS page, showing the differential expression of the RNA components between condensates and controls.

All data deposited in RPS are available on the ‘Download’ page. Detailed guidance on the usage of RPS can be found on the ‘Help’ page.

## SUMMARY AND PERSPECTIVES

Phase separation has expanded our understanding of biochemical reactions and biological processes in MLOs. With the advancement of phase separation research technology, more and more RNAs have been found to participate in LLPS process and regulate the assembly of MLOs. Emerging evidence has shown that RNA is crucial in sensing stress stimulations, signal transduction, and maintenance of phase separation. Despite that, existed LLPS-associated databases primarily focus on proteins (13–18), while a comprehensive, curated database of LLPS-related RNAs is still lacking. To our knowledge, RPS is the first comprehensive database for specifically hosting the LLPS-related RNAs. It records both basic information of all LLPS-related RNAs and details of the corresponding LLPS system, such as phase behavior and experimental conditions. For humans, mice and yeast, RPS offers additional annotations of RNA features that confer condensate biophysical properties, including RNA structures, RNA-associated interactions and modifications. Multiple comparisons of these RNA features between LLPS-related RNAs and non-LLPS-related RNAs revealed that RNA sequence, m^6^A modifications, as well as multivalent interactions were likely to promote LLPS (Figure 2A–D). These observations are in line with previous findings (1,23,63,64), illustrating the ability of RPS to reveal the contribution of RNAs in mediating LLPS. Remarkably, LLPS-related RNAs exhibit a stronger correlation with disease than non-LLPS-related RNAs (Figure 2E), suggesting that a deep understanding of the contribution of RNA to LLPS will be beneficial for disease diagnosis and treatment.

Taken together, we anticipate that RPS will support investigations into the potential of RNA in condensate biology and develop better therapeutic treatments for phase-separation-related diseases. With the growing interest in the role of RNA in LLPS, there will be a rapidly increasing number of relevant studies and more LLPS-related RNAs will be discovered. Therefore, we are dedicated to ensuring the long-term maintenance and reliability of the RPS database by continuous updates and careful validation.

## DATA AVAILABILITY

RPS is a comprehensive online database available at http://rps.renlab.org.

## Supplementary Material

gkab986_Supplemental_File
